# Temporal mTOR inhibition protects Fbxw7-deficient mice from radiation-induced tumor development

**DOI:** 10.18632/aging.100535

**Published:** 2013-02-24

**Authors:** Yueyong Liu, Yurong Huang, Zeran Wang, Yong Huang, Xiaohua Li, Alexander Louie, Guangwei Wei, Jian-Hua Mao

**Affiliations:** ^1^ Life Sciences Division, Lawrence Berkeley National Laboratory, Berkeley, CA 94720, USA; ^2^ Drug Studies Unit, University of California at San Francisco, South San Francisco, CA 94080, USA; ^3^ Department of Anatomy, Shandong University School of Medicine, Jinan, Shandong, 250012 China

**Keywords:** Fbxw7, mTOR, p53, radiation, tumorigenesis, rapamycin

## Abstract

*FBXW7* acts as a tumor suppressor in numerous types of human cancers through ubiquitination of different oncoproteins including mTOR. However, how the mutation/loss of *Fbxw7* results in tumor development remains largely unknown. Here we report that downregulation of mTOR by radiation is *Fbxw7*-dependent, and short-term mTOR inhibition by rapamycin after exposure to radiation significantly postpones tumor development in *Fbxw7*/*p53* double heterozygous (*Fbxw7*+/−*p53*+/−) mice but not in *p53* single heterozygous (*p53*+/−) mice. Tumor latency of rapamycin treated *Fbxw7*+/−*p53*+/− mice is remarkably similar to those of *p53*+/− mice while placebo treated *Fbxw7*+/−*p53*+/− mice develop tumor significantly earlier than placebo treated *p53*+/− mice. Furthermore, we surprisingly find that, although temporal treatment of rapamycin is given at a young age, the inhibition of mTOR activity sustainably remains in tumors. These results indicate that inhibition of mTOR signaling pathway suppresses the contribution of *Fbxw7* loss toward tumor development.

## INTRODUCTION

*FBXW7* is one of the most important human tumor suppressor genes, which undergoes deletion and/or mutation in cancers from a wide spectrum of human tissues, such as breast, colon, endometrium, stomach, lung, ovary, pancreas, and prostate [1, 2]. The overall frequency of point mutation of *FBXW7* in human cancers is about 6% [3]. The *FBXW7* gene is essential for the ubiquitination of different oncoproteins, including c-Myc [4, 5], c-Jun [6], cyclin E [7, 8], Notch [9, 10], Klf5 [11, 12], Mcl-1 [13, 14], and Aurora-A [15, 16]. Haploinsufficient loss of *Fbxw7* is observed in most lymphomas in the mouse model, even those arising from *Fbxw7/p53* double heterozygous mice [17]. Similar observations of heterozygous mutations were subsequently made in human tumors [18]. These findings suggest that loss of only one copy of the gene can generate a substantial biological impact.

The mammalian target of rapamycin, mTOR, is a central component of several complex signaling networks that regulate cell growth, metabolism and proliferation. mTOR signaling is frequently dysregulated in a number of human diseases including cancer, cardiovascular disease and ageing, and thus has become an attractive target for therapeutic intervention. We and others have recently shown that mTOR is a target of FBXW7 [19-21]. In this study, we investigated whether inhibition of mTOR signaling pathway by rapamycin was able to prevent the tumor development resulted from loss of Fbxw7 in mice.

## RESULTS

### Fbxw7-dependent inhibition of mTOR by radiation

**Figure 1:**
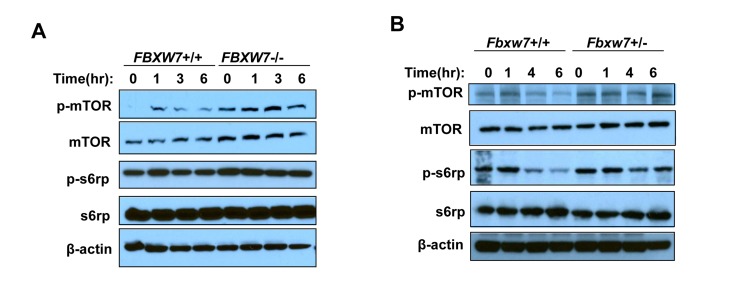
Radiation inhibits mTOR and its signaling in a FBXW7-depentend manner. mTOR and its signaling was assessed by Western blot assays with antibodies to p-mTOR (Ser2448), mTOR, p-S6rp (Ser240 and Ser244), S6rp, and β-Actin. (**A**) Detection of mTOR and its downstream signaling in HCT116 wild type and *FBXW7*−/− cells at different time points after single dose of 4Gy X-ray radiation. (**B**) Detection of mTOR and its downstream signaling in thymuses from wild type and *FBXW7*+/− mice that were collected at different time points after single dose of 4Gy X-ray radiation.

Our previous study has shown that *Fbxw7* can be transcriptionally activated by *p53* upon DNA damage [17]. Thus we first sought to investigate the changes in mTOR signaling pathway after exposure to radiation. Western blot analysis showed that, at different time points post radiation, there is a decrease in the phosphorylation levels of mTOR (p-mTOR) in HCT116 *FBXW7*+/+ cells while there is no change in HCT116 *FBXW7*−/− cells, which is confirmed by downstream the phosphorylation levels of s6 Ribosomal Protein (p-s6rp) (Fig. 1A). We also observed a significant increase in mTOR and p-mTOR level in HCT116 *FBXW7*−/− cells compared to HCT116 *FBXW7*+/+ cells, consistent with our previous report [19]. These observations were further examined using *Fbxw*7 wild-type (*Fbxw*7+/+) and heterozygous (*Fbxw*7+/−) mice (Fig. 1B). Loss of one copy of *Fbxw7* sufficiently blocked the radiation-induced decrease in level of total mTOR and p-mTOR (Fig. 1B). All these results clearly indicate that inhibition of mTOR and its signaling by radiation is FBXW7-dependent.

### Temporal rapamycin treatment delays tumorigenesis in *Fbxw7*/*p53* double heterozygous (*Fbxw7*+/−*p53*+/−) mice, not in *p53* single heterozygous (*p53*+/−) mice

**Figure 2:**
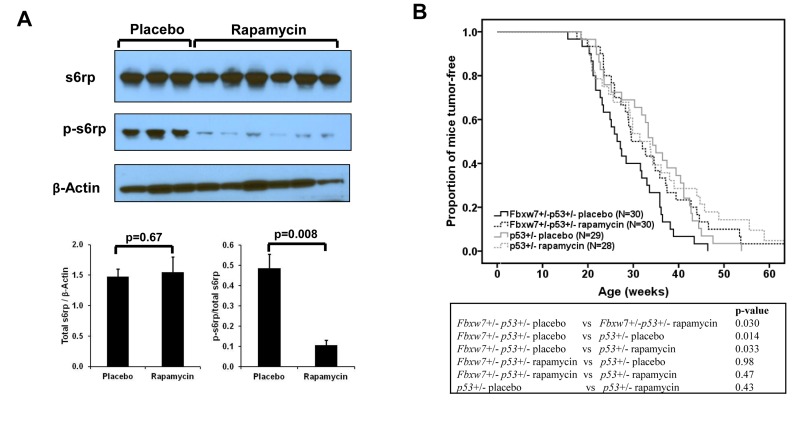
Effect of rapamycin on mTOR signaling and radiation-induced tumor development. (**A**) Western blotting and quantitative analysis of the blots shows decreased p-s6rp (Ser240 and Ser244) level in spleen when mice treated with rapamycin. No change was found in total s6rp. Mean values (± standard deviation) were presented. The p-values were obtained by t-test. (**B**) Radiation-induced tumorigenesis in *Fbxw7*+/−*p53*+/− or *p53*+/− mice with 10-week treatment of rapamycin or placebo that was given at 1 week post a single dose of 4Gy X-ray radiation. Top panel: Kaplan-Meier curves of tumor latency. Bottom panel: The p-values were obtained from long rank test by Kaplan-Meier analysis.

Next, we investigated whether temporal mTOR inhibition by rapamycin can prevent mice from *Fbxw7* loss-induced tumor development. We decided upon administration using 10-week continuous release pellets embedded with rapamycin (at dose of 4mg/kg body weight/day) to standardize rapamycin treatment. First we examined blood levels of rapamycin in the treated mice with this pellet at different time points using liquid chromatography-tandem mass spectrometry (details see Materials and Methods). We observed that in rapamycin-treated mice the average rapamycin level was about 20ng/ml and could not be detected at 15 weeks after pellet implantation, whereas in placebo-treated mice rapamycin concentration was always below the detection level (Supplementary Fig. S1). Next we assessed the biochemical effects of rapamycin by measuring the levels of p-s6rp in spleen. Western blotting analysis showed that the levels of total s6rp were similar between placebo and rapamycin treated groups (Fig. 2A). In contrast, we found that rapamycin reduced the levels of p-s6rp (Fig. 2A), suggesting that the kinase activity of mTOR was inhibited in the rapamycin-treated mice in comparison to the placebo-treated mice.

In order to investigate whether rapamycin can prevent mice from *Fbxw7* loss-induced tumor development, 60 *Fbxw7*+/−*p53*+/− mice were treated with a single dose of 4Gy whole body X-Ray irradiation at about 5-week old and were randomly divided into two groups (Supplementary Table S1). One week after irradiation, one group was treated with the 10-week continuous release rapamycin pellets and the other group was treated with placebo pellets (details see Materials and Methods). As a control, 57 *p53*+/− mice were treated using the same protocol (Supplementary Table S1). We found that, in *Fbxw7*+/−*p53*+/− mice, temporal rapamycin treatment significantly delayed the tumor development (p=0.03) (Fig. 2B). In contrast, such temporal rapamycin treatment is ineffective in *p53*+/− mice (p=0.43), although showing a trend toward delay in tumor development in late life (Fig. 2B). Consistent with our previous finding [17], placebo-treated *Fbxw7*+/−*p53*+/− mice developed tumors much earlier than p53+/− mice (p=0.014) (Figure 2b). Strikingly, rapamycin-treated *Fbxw7*+/−*p53*+/− mice were equivalent to p53+/− mice in radiation sensitivity (Fig. 2B). Furthermore, the tumor spectra between placebo- and rapamycin-treated mice are similar (Supplementary Fig. S2). These results suggested that temporal rapamycin treatment fully blocked the contribution of Fbxw7 loss to radiation-induced tumor development.

### Sustained inactivation of mTOR signaling pathway in tumors from mice with temporal rapamycin treatment

**Figure 3:**
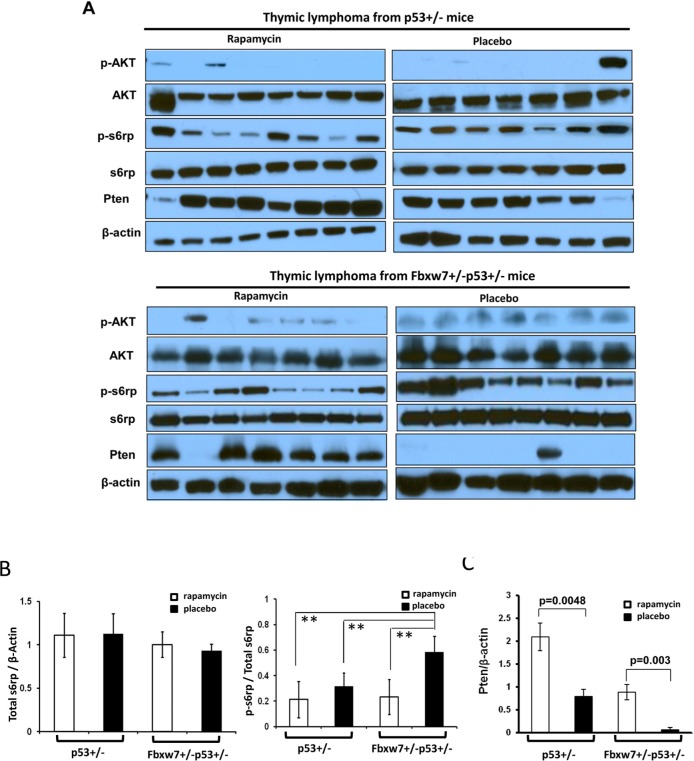
Inhibition of mTOR signaling sustains in tumors from rapamycin treated mice. (**A**) Detection of mTOR upstream and downstream signaling in the tumors from *Fbxw7*+/−*p53*+/− and *p53*+/− mice treated with rapamycin or placebo by Western blot assays with antibodies to p-AKT (Ser473), AKT, p-S6rp (Ser240 and Ser244), S6rp, Pten, and β-Actin. (**B**) Quantitative analysis of the total s6rp and p-s6rp levels in the blots showed in (**A**). Mean values (± standard deviation) were presented. **indicates p<0.001. (**C**) Quantitative analysis of the Pten levels in the blots showed in (A). Mean values (± standard deviation) were presented.

Next we investigated the effects of temporal rapamycin treatment on mTOR signaling in the tumor tissues by Western blotting. Although there was no difference in the levels of total s6rp among different genotype and treatment groups (p=0.13) (Fig. 3), one consistent observation was that tumors from rapamycin treated *Fbxw7*+/−*p53*+/− mice retained the significantly lower average levels of p-s6rp in comparison to those from placebo treated *Fbxw7*+/−*p53*+/− mice (p<0.001) (Fig. 3A and B). There are slightly lower average levels of p-s6rp in tumors from rapamycin treated *p53*+/− mice than in those from placebo treated *p53*+/− mice, but not significant difference (p=0.12) (Fig. 3B). Interestingly, tumors from rapamycin treated *Fbxw7*+/−*p53*+/− mice showed a similar range of p-s6rp levels as those from rapamycin treated *p53*+/− mice while tumors from placebo treated *Fbxw7*+/−*p53*+/− mice showed significantly higher p-s6rp levels than these from placebo treated *p53*+/− mice (p<0.001) (Fig. 3B), suggesting mTOR activity is elevated when loss of one copy of *Fbxw7*, and this elevation is inhibited by mTOR inhibitor, rapamycin. Presumably such inhibition by rapamycin subsequently suppresses the contribution of Fbxw7 loss to tumor development.

Another interesting finding was that the protein level of Pten had a significant increase in tumors from rapamycin treated *p53*+/− and *p53*+/−*Fbxw7*+/− mice (Fig. 3C). Especially in those placebo treated *p53*+/−*Fbxw7*+/− mice, Pten level was only detected in one of eight tumors. This observation was confirmed by immunochemical staining in tumors (Supplementary Fig. S3). The possible explanation for this is due to the complex feedback loops in mTOR pathway that has been reported [22].

## DISCUSSION

Our results demonstrate that mTOR signaling pathway is inhibited following radiation exposure, which can be explained by that radiation activates p53, in turn p53 transcriptionally upregulates Fbxw7, subsequently Fbxw7 downregulates mTOR through ubiquitination. p53 inhibits mTOR through Fbxw7 and subsequently prevents cellular senescence [23-25]. This explanation is supported by that depletion of *Fbxw7* blocks radiation-induced mTOR inhibition. Interestingly, a recent study shows that PI3K-AKT-mTOR signaling pathway is activated in mouse mammary gland at 2 and 12 months post radiation exposure [26], suggesting that (a) mTOR signaling in different tissues possibly responses to radiation differently since we examined mTOR signaling in thymuses; and (b) long-term effect of radiation on mTOR signaling is possibly different from short-term one. Additional experiments are required to clarify this difference by systematic assessment of mTOR signaling in different tissues and at different time points and to examine the mechanisms underlying these different responses. It is possible that radiation modulates mTOR signaling via p53-Fbxw7 pathway at earlier time point whereas via different pathway(s) at long-term post exposure.

Fbxw7 regulates mTOR via its ubiquitination function [19-21]. Depletion of Fbxw7 leads to elevation of mTOR signaling, which drives many cell growth outputs. Thus we assume that inhibition of mTOR activity by rapamycin may act a major brake on tumor development in Fbxw7 deficient mice. Indeed, *Fbxw7*+/−*p53*+/− mice with temporal rapamycin treatment after radiation develop tumor same as *p53*+/− mice, while *Fbxw7*+/−*p53*+/− mice with temporal placebo treatment develop tumor significantly faster than *p53*+/− mice. Even more, tumors from rapamycin treated *Fbxw7*+/−*p53*+/− mice showed similar mTOR signaling as those from rapamycin treated *p53*+/− mice while tumors from placebo treated *Fbxw7*+/−*p53*+/− mice showed significantly higher mTOR signaling than these from placebo treated *p53*+/− mice. These results indicate that rapamycin inhibits mTOR signaling pathway and in turn, such inhibition fully suppresses the contribution of *Fbxw7* loss toward tumor development.

We observed that the same temporal inhibition of mTOR pathway could not sufficiently prevent p53+/− mice from radiation-induced tumor development. This observation is different from the recent report about anti-cancer effect of rapamycin in p53+/− mice [27] and that cellular senescence of normal cells predispose to cancer [28, 29]. This is difference is possibly due to rapamycin treatment regimen. In their study, p53+/− mice were continuously treated with rapamycin beginning at a young age (<5 months) whereas we temporally treated p53+/− mice with rapamycin at age about 1.5 through 4 months. Other possible reason is that they did not use radiation, tumor were spontaneous. In our study, mice were irradiated at 5 weeks old, and rapamycin treatment was given at 1 week after radiation. It is possible that we missed the window of prevention since it has been reported that rapamycin is better for prevention than treatment [29].

In conclusion, *FBXW7* has emerged as a major human tumor suppressor gene that lies at the nexus of several pathways which control cell growth, cell differentiation, and tumorigenesis, including those mediated by Ras, Myc, Jun, p53, Notch and mTor. How the decrease in Fbxw7 function results in tumor development remains largely unknown. Mutation/loss of the *Fbxw7* gene may cause impaired degradation of multiple targets, and as a result constitutive accumulation of these targets may cooperatively contribute to tumor development. Our results in this study showed that temporal pharmacological inhibition of mTOR pathway after radiation was sufficient to suppress the tumor development contributed by *Fbxw7* loss, suggesting that Fbxw7-mTOR pathway plays a major role in this radiation-induced carcinogenesis mouse model.

## MATERIALS AND METHODS

### Mice, tumor induction, and rapamycin treatment

*Fbxw7*+/− mice was crossed with *p53*−/− mice to generate *p53*+/− and *p53*+/−*Fbxw7*+/− mice. The 5-week old *p53*+/− and *p53*+/−*Fbxw7*+/− mice were exposed to a single dose of 4Gy whole body X-ray irradiation. One week after radiation treatment, mice were randomly divided into two groups. One group of mice was treated with rapamycin, the other with placebo. The treatment was administrated by subcutaneously implanting the 10-week continuous release pellets embedded with rapamycin or placebo. The rapamycin and placebo pellet were purchased from Innovative Research of America (Sarasota, Florida USA. Website: http://www.innovrsrch.com). The rapamycin pellet released at a dose of 4mg/kg/day based upon the average mouse weight of 20g. Mice were observed daily until moribund, then euthanized and autopsied. Mice were bred and treated under the protocol approved by Animal Welfare and Research Committee at Lawrence Berkeley National Laboratory.

### Measurement of Rapamycin concentration in blood

Whole blood was collected from rapamycin or Placebo treated mice by retro-orbital or tail vein bleeding into EDTA tubes and stored at −70°C until analysis. Rapamycin was measured by liquid chromatography-tandem mass spectrometry (LC/MS/MS). The standard curve range for rapamycin was 1ng/ml to 400ng/ml. The standard curve samples were made by spiking blank blood with different amounts of rapamycin and processed along with the study samples. The blood sample (20μl) was diluted with 20μl of water and then 40μl of 70% acetonitrile was added. 20μl of internal standard, rapamycin-d3 (10ng/ml), was added to each sample. 100μl of methanol: 0.3M zinc sulfate (70:30) (*v/v*) was added and vortexed for 1min. The mixture was centrifuged at 3000 rpm for 10 min. Then the supernatant was transferred to an autosampler vial and 5μl was injected to the following LC/MS/MS system. The mass detector was an API 5000 triple quadrapole (Applied Biosystems, Foster City, CA), equipped with a Turbo Ion Spray source. The system was set in positive ionization mode. The ion spray voltage was 5500V and the source temperature was 400°C. The values for CAD, CUR, GS1, and GS2 were 8, 20, 75, and 75 respectively. The multiple reaction monitor was set at 931.8 − 864.7 *m/z* for rapamycin and 934.8 − 864.7 *m/z* for Sirolimus-d3. The values for DP, EP, CE, CXP were 80, 10, 22, and 45 respectively for rapamycin and Sirolimus-d3. A Shimadzu system was used for the HPLC, consisting of a pump, solvent degasser, autosampler and column oven. The column oven was set to 50°C and the autosampler was set to 4°C. The mobile phase, consisting of 65 % acetonitrile, 0.05 % formic acid containing 1mM ammonium acetate, was pumped through a Hypersil BDS C8 (3 × 50 mm, 5 μm particle size) column with a flow rate of 0.40 ml/min. Data was acquired and processed by Analyst 1.5.1 software.

### Antibody and Immunoblotting

Western blot assays were performed with antibodies to phospho-mTOR (Ser2448), mTOR, phospho-S6 ribosomal protein at Ser240 and S244 (p-s6rp), s6 ribosomal protein (s6rp), phospho-AKT (S473), AKT, Pten, and beta-Actin. All antibodies were purchased from Cell Signaling Technology (Danvers, MA, USA).

Spleen tissue was dissected from mice that had been implanted a rapamycin pellet for 5 weeks. Thymic lymphomas were collected and stored at −80°c. Tissues were minced by blue pestle using M-PER lysis buffer (Pierce) supplemented with protease inhibitor cocktail (Roche), 10μM phenylmethylsulfonyl fluoride,and 1 mM sodium orthovanadate. Protein extract was separated on 10% SDS-PAGE electrophoresis gels. Proteins were transferred to Hybond P membranes (Amersham, Piscataway, NJ). Nonspecific bands were blocked in 5% non-fat milk for 1 hour at room temperature and then in appropriate primary antibody overnight at 4°C. After incubating with a horseradish peroxidase-linked secondary antibody, proteins were visualized by enhanced chemiluminescence (Amersham). Images were digitally acquired using an HP ScanJet 5200C Scanner and quantified using AlphaEaseFC image analysis software.

### Statistical Analysis

Comparison of Pten level, total s6rp and p-s6rp levels in either normal tissues or thymic lymphomas between treatment and genotype groups was carried out by the two-tailed Student's t test or ANOVA. The Kaplan–Meier method was used to compare the tumor development after irradiation of mice between different treatments and genotypes. Statistical analysis was performed using SPSS version 12.0 (SPSS, Chicago, IL).

## SUPPLEMENRATY DATA



## References

[R1] Welcker M, Clurman BE (2008). FBW7 ubiquitin ligase: a tumour suppressor at the crossroads of cell division, growth and differentiation. Nat Rev Cancer.

[R2] Cheng Y, Li G (2012). Role of the ubiquitin ligase Fbw7 in cancer progression. Cancer Metastasis Rev.

[R3] Akhoondi S, Sun D, von der Lehr N, Apostolidou S, Klotz K, Maljukova A, Cepeda D, Fiegl H, Dafou D, Marth C, Mueller-Holzner E, Corcoran M, Dagnell M (2007). FBXW7/hCDC4 is a general tumor suppressor in human cancer. Cancer Res.

[R4] Welcker M, Orian A, Grim JE, Eisenman RN, Clurman BE (2004). A nucleolar isoform of the Fbw7 ubiquitin ligase regulates c-Myc and cell size. Curr Biol.

[R5] Yada M, Hatakeyama S, Kamura T, Nishiyama M, Tsunematsu R, Imaki H, Ishida N, Okumura F, Nakayama K, Nakayama KI (2004). Phosphorylation-dependent degradation of c-Myc is mediated by the F-box protein Fbw7. Embo J.

[R6] Wei W, Jin J, Schlisio S, Harper JW, Kaelin WG (2005). The v-Jun point mutation allows c-Jun to escape GSK3-dependent recognition and destruction by the Fbw7 ubiquitin ligase. Cancer Cell.

[R7] Koepp DM, Schaefer LK, Ye X, Keyomarsi K, Chu C, Harper JW, Elledge SJ (2001). Phosphorylation-dependent ubiquitination of cyclin E by the SCFFbw7 ubiquitin ligase. Science.

[R8] Rajagopalan H, Jallepalli PV, Rago C, Velculescu VE, Kinzler KW, Vogelstein B, Lengauer C (2004). Inactivation of hCDC4 can cause chromosomal instability. Nature.

[R9] Gupta-Rossi N, Le Bail O, Gonen H, Brou C, Logeat F, Six E, Ciechanover A, Israël A (2001). Functional interaction between SEL-10, an F-box protein, and the nuclear form of activated Notch1 receptor. J Biol Chem.

[R10] Oberg C, Li J, Pauley A, Wolf E, Gurney M, Lendahl U (2001). The Notch intracellular domain is ubiquitinated and negatively regulated by the mammalian Sel-10 homolog. J Biol Chem.

[R11] Liu N, Li H, Li S, Shen M, Xiao N, Chen Y, Wang Y, Wang W, Wang R, Wang Q, Sun J, Wang P (2010). The Fbw7/human CDC4 tumor suppressor targets proproliferative factor KLF5 for ubiquitination and degradation through multiple phosphodegron motifs. J Biol Chem.

[R12] Zhao D, Zheng HQ, Zhou Z, Chen C (2010). The Fbw7 tumor suppressor targets KLF5 for ubiquitin-mediated degradation and suppresses breast cell proliferation. Cancer Res.

[R13] Inuzuka H, Shaik S, Onoyama I, Gao D, Tseng A, Maser RS, Zhai B, Wan L, Gutierrez A, Lau AW, Xiao Y, Christie AL, Aster J (2011). SCF(FBW7) regulates cellular apoptosis by targeting MCL1 for ubiquitylation and destruction. Nature.

[R14] Wertz IE, Kusam S, Lam C, Okamoto T, Sandoval W, Anderson DJ, Helgason E, Ernst JA, Eby M, Liu J, Belmont LD, Kaminker JS, O'Rourke KM (2011). Sensitivity to antitubulin chemotherapeutics is regulated by MCL1 and FBW7. Nature.

[R15] Fujii Y, Yada M, Nishiyama M, Kamura T, Takahashi H, Tsunematsu R, Susaki E, Nakagawa T, Matsumoto A, Nakayama KI (2006). Fbxw7 contributes to tumor suppression by targeting multiple proteins for ubiquitin-dependent degradation. Cancer Sci.

[R16] Kwon YW, Kim IJ, Wu D, Lu J, Stock WA, Liu Y, Huang Y, Kang HC, DelRosario R, Jen KY, Perez-Losada J, Wei G, Balmain A, Mao JH (2012). Pten regulates aurora-a and cooperates with fbxw7 in modulating radiation-induced tumor development. Mol Cancer Res.

[R17] Mao JH, Perez-Losada J, Wu D, Delrosario R, Tsunematsu R, Nakayama KI, Brown K, Bryson S, Balmain A (2004). Fbxw7/Cdc4 is a p53-dependent, haploinsufficient tumour suppressor gene. Nature.

[R18] Kemp Z, Rowan A, Chambers W, Wortham N, Halford S, Sieber O, Mortensen N, von Herbay A, Gunther T, Ilys M, Tomlinson I (2005). CDC4 mutations occur in a subset of colorectal cancers but are not predicted to cause loss of function and are not associated with chromosomal instability. Cancer Res.

[R19] Balamurugan K, Wang JM, Tsai HH, Sharan S, Anver M, Leighty R, Sterneck E (2010). The tumour suppressor C/EBPdelta inhibits FBXW7 expression and promotes mammary tumour metastasis. Embo J.

[R20] Fu L, Kim YA, Wang X, Wu X, Yue P, Lonial S, Khuri FR, Sun SY (2009). Perifosine inhibits mammalian target of rapamycin signaling through facilitating degradation of major components in the mTOR axis and induces autophagy. Cancer Res.

[R21] Mao JH, Kim IJ, Wu D, Climent J, Kang HC, DelRosario R, Balmain A (2008). FBXW7 targets mTOR for degradation and cooperates with PTEN in tumor suppression. Science.

[R22] Beauchamp EM, Platanias LC (2012). The evolution of the TOR pathway and its role in cancer. Oncogene.

[R23] Leontieva OV, Blagosklonny MV (2010). DNA damaging agents and p53 do not cause senescence in quiescent cells, while consecutive re-activation of mTOR is associated with conversion to senescence. Aging (Albany NY).

[R24] Korotchkina LG, Leontieva OV, Bukreeva EI, Demidenko ZN, Gudkov AV, Blagosklonny MV (2010). The choice between p53-induced senescence and quiescence is determined in part by the mTOR pathway. Aging (Albany NY).

[R25] Demidenko ZN, Korotchkina LG, Gudkov AV, Blagosklonny MV (2010). Paradoxical suppression of cellular senescence by p53. Proc Natl Acad Sci U S A.

[R26] Suman S, Johnson MD, Fornace AJ, Datta K (2012). Exposure to ionizing radiation causes long-term increase in serum estradiol and activation of PI3K-Akt signaling pathway in mouse mammary gland. Int J Radiat Oncol Biol Phys.

[R27] Komarova EA, Antoch MP, Novototskaya LR, Chernova OB, Paszkiewicz G, Leontieva OV, Blagosklonny MV, Gudkov AV (2012). Rapamycin extends lifespan and delays tumorigenesis in heterozygous p53+/− mice. Aging (Albany NY).

[R28] Mercier I, Camacho J, Titchen K, Gonzales DM, Quann K, Bryant KG, Molchansky A, Milliman JN, Whitaker-Menezes D, Sotgia F, Jasmin JF, Schwarting R, Pestell RG, Blagosklonny MV, Lisanti MP (2012). Caveolin-1 and accelerated host aging in the breast tumor microenvironment: chemoprevention with rapamycin, an mTOR inhibitor and anti-aging drug. Am J Pathol.

[R29] Blagosklonny MV (2012). Rapalogs in cancer prevention: Anti-aging or anticancer?. Cancer Biol Ther.

