# Supplementary material for: Temporal mTOR inhibition protects Fbxw7-deficient mice from radiation-induced tumor development
Source: Aging (Albany NY). 2013 Feb 24;5(2):111–9. doi: 10.18632/aging.100535 (PMC3616198; doi:10.18632/aging.100535)
Supplement: Supplementary file 1 [file aging-05-111-s001.pdf]

SUPPLEMENRATY DATA

Supplementary Table S1. Number of mice in different genotype and treatment groups

Genotype	Treatment group	Gender		Total
		Male	Female	
p53+/-Fbxw7+/-	Rapamycin	15	15	30
	Placebo	15	15	30
p53+/-Fbxw7+/+	Rapamycin	14	15	29
	Placebo	14	14	28

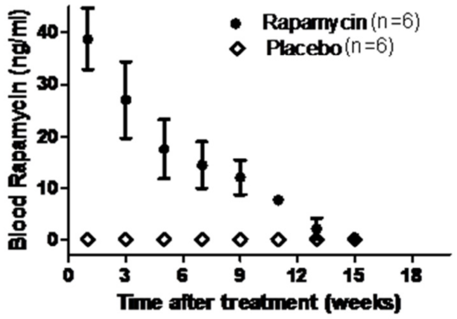

Supplementary Figure S1. Rapamycin levels in blood were measured by liquid chromatography-tandem mass spectrometry (LC/MS/MS) at different time points after rapamycin pellet implantation.

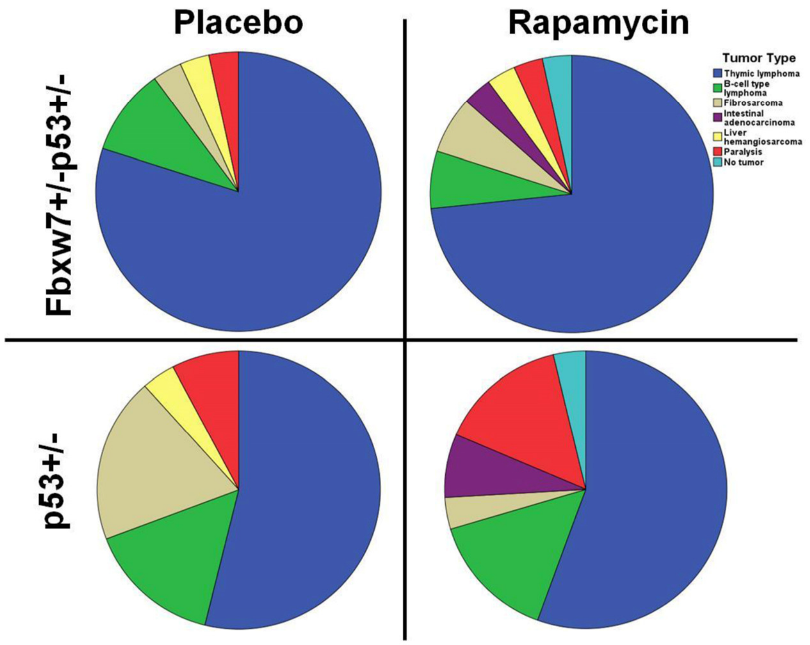

Supplementary Figure S2. Tumor spectrum in placebo or rapamycin treated Fbxw7+/- p53+/- or p53+/- mice.

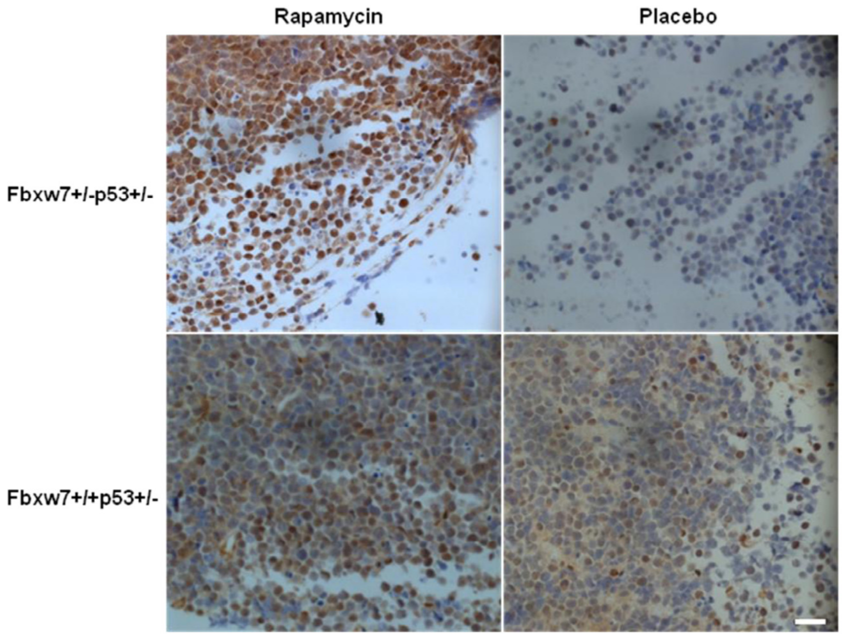

Supplementary Figure S3. Immunohistochemical staining of Pten in tumor from placebo or rapamycin treated Fbxw7+/- p53+/- mice.
